# Significant Difference in Acute Coronary Lesions in Patients on Therapeutic Statin Therapy Compared to Those Without

**DOI:** 10.7759/cureus.10673

**Published:** 2020-09-26

**Authors:** Chikezie Alvarez, Ernestine Keh, Yiting Li, Henry Siu

**Affiliations:** 1 Cardiology, Hartford Hospital/University of Connecticut, Hartford, USA; 2 Internal Medicine, St. Francis Medical Center, Trenton, USA; 3 Interventional Cardiology, Chinatown Cardiology, P.C., New York, USA

**Keywords:** acute coronary syndrome, statin therapy, in-stent restenosis, stent thrombosis, plaque morphology

## Abstract

Background

Statin therapy has been shown to alter coronary plaque via decreasing lipid-rich pools, substituting with fibrocalcific plaque. We propose that acute coronary lesions in patients on therapeutic statin therapy, compared with patients not on statin therapy with elevated low-density lipoprotein (LDL) levels, will have features typically not associated with lipid-rich disease, such as lengthy fibrocalcific lesions, stent failure (in-stent restenosis and stent thrombosis), and/or bypass graft degeneration.

Methods

Charts and coronary angiograms of 143 consecutive patients from May 2016 to December 2018 with Type 1 Myocardial Infarction Fourth Universal Definition were retrospectively reviewed. Patients were divided into two groups: group 1, those on statin therapy with an LDL < 100 mg/dL, and group 2, those not on statin therapy with an LDL ≥ 100 mg/dL at the time of an acute coronary syndrome. Acute lesion characteristics and angioplasty techniques/equipment were recorded.

Results

There were 56 patients in group 1 and 39 patients in group 2. Group 1 patients, compared with group 2, were more likely to have moderate-severe vessel calcification (55.4% vs 10.3%; p < 0.01), lesion length greater than 20 mm (51.8% vs 23.1%; p = 0.019), stent failure (39.3% vs 7.7%; p < 0.01), and bypass graft failure (23.2% vs 7.7%; p = 0.04). Coronary interventions in group 1, compared with group 2, more often required adjunctive angioplasty techniques, including the need for multiple coronary wires, guide extender, thrombectomy, and circulatory support (51.8% vs 7.7%; p < 0.01). There was no significant difference between the number of culprit vessels, including the specific coronary culprit vessel identified between both groups.

Conclusion

Acute coronary lesions in patients on therapeutic statin therapy, compared to those not on statin therapy with an elevated LDL, tend to be longer, more calcific with increased vessel tortuosity and angulation, as well as from a stent or bypass graft failure mechanism. Furthermore, there is a signal for heightened procedural complexity in the arm with statin therapy. These clinical findings are likely the result of the altered natural history of plaque pathophysiology seen with statin therapy.

## Introduction

Acute coronary syndromes classically originate from plaque rupture of lipid core/rich plaques. A smaller portion is a consequence of calcified plaques and plaque erosion [1]. A non-inconsequential portion of acute coronary pathologies are a result of other mechanisms, including stent failure involving in-stent restenosis or stent thrombosis, or via coronary bypass graft failures.

Lipid-lowering therapy with statins has been recognized to change plaque morphology [2]. On a histological level, plaques subjected to statin therapy become void of lipid cores, and diseased vessels demonstrate less thin-capped fibroatheromas (TCFAs) over time [3]. In turn, efficacious statin therapy can be implicated in altering the natural pathophysiological history of acute plaque progression and clinical events. ﻿

With statin therapy as the foundation of primary and secondary cardiovascular prevention, there can be a shift in the presentation of the acute coronary lesion phenotype to one that is not undermined by lipid-rich plaques and TCFAs. These “residual” acute coronary pathologies, in particular calcified lesions and revascularization failures, are hypothesized to be prevalent in statin-treated patients. This study intents to elucidate and characterize this potential landscape change in the presentation of acute coronary lesions.

## Materials and methods

Setting

This retrospective single center observational study was conducted at Saint Francis Medical Center in Trenton, New Jersey (USA). This study was approved by our centers’ ﻿Institutional Review Board.

Study population and design

Charts and coronary angiograms of 143 consecutive patients from May 2016 to December 2018 with Fourth Universal Definition Type 1 Myocardial Infarction (MI) were retrospectively reviewed [4]. In depth chart review for exclusion of non-type 1 MI was performed (i.e. troponin elevation due to congestive heart failure, tachyarrhythmias, or other causes of oxygen-demand mismatch).

Patients were stratified into two groups: group 1, those on statin therapy with an low-density lipoprotein (LDL) < 100 mg/dL, and group 2, those not on statin therapy with an LDL ≥ 100 mg/dL at the time of an acute coronary syndrome. Of the 143 patients, 56 patients fit the criteria for group 1 and 39 patients for group 2. A total of 48 patients were not included in the final analysis; they were either (1) on statin therapy with an LDL ≥ 100 mg/dL or (2) not on statin therapy with an LDL < 100 mg/dL at the time of an acute coronary syndrome.

Baseline patient and laboratory characteristics were recorded, including age, gender, active tobacco use, medical comorbidities, lipid profile, and antiplatelet and/or anticoagulant use. Acute lesions, coronary vessel characteristics, and angioplasty techniques/equipment used were analyzed.

Statistical analysis

﻿Continuous data were represented as mean ± SD. Categorical data were expressed as numbers and percentages. Data were tested for normality using the Shapiro-Wilk test, and examination of q-q plots. Comparison of continuous variables between the two groups was performed using the unpaired independent samples Student's t test. Univariate categorical data were evaluated for significance by Fisher's exact test when the expected frequency was <5 and chi-squared testing in all other cases. P < 0.05 was considered to indicate a statistically significant result. Statistical analysis was done using SPSS version 26.0 software (SPSS Inc., Chicago, IL, USA).

## Results

Baseline patient characteristics and laboratory data

Baseline characteristics are summarized in Tables 1, 2, and were similar between group 1 and group 2 with regards to gender (69.6% vs 71.8% male), age (70.4 ± 8.7 vs 67.1 ± 11.1), active tobacco use (50% vs 51.3%), body mass index (mean 28.8 vs 30.5), and history of diabetes mellitus (p = 0.14). At baseline, aspirin (p < 0.01), P2Y12 inhibitor (p < 0.01), and oral anticoagulant use (p = 0.02) were significantly greater in the therapeutic statin therapy group compared to the non-statin group. There was no significant difference between the two groups regarding a history of coronary artery bypass graft (CABG) prior to presentation (30.4% vs 17.9% p = 0.17).

**Table 1:** Baseline Patient Characteristics Between Therapeutic Statin Group vs No Statin Therapy Values are n (%) or mean ± SD. Chi-squared test was used for categorical data, and unpaired Student's t test was used for continuous data between group 1 and group 2. Values of p < 0.05 were considered statistically significant. BMI, body mass index; HTN, hypertension; DM, diabetes mellitus; CKD, chronic kidney disease; CAD, coronary artery disease; CABG, coronary artery bypass graft.

	Therapeutic Statin (Group 1)	No Statin (Group 2)	P Value
n	56	39	
Age	70.4 ± 8.75	67.1 ± 11.1	0.11
Male	39 (69.6)	28 (71.8)	0.82
Race			
White	39 (69.7)	23 (59)	0.53
African American	13 (23.2)	13 (33.3)	
Hispanic	4 (7.1)	3 (7.7)	
Active tobacco use	28 (50)	20 (51.3)	0.90
BMI	28.8 ± 6.25	30.5 ± 4.62	0.16
History of HTN	51 (91.1)	25 (64.1)	<0.01
History of DM	30 (53.6)	15 (38.5)	0.14
History of CKD	10 (17.9)	2 (5.1)	0.06
History of CAD	37 (66.1)	13 (33.3)	0.002
History of CABG	17 (30.4)	7 (17.9)	0.17
Aspirin	46 (82.1)	9 (23.1)	<0.01
P2Y12 inhibitor	23 (41.1)	3 (7.7)	<0.01
Oral anticoagulant	7 (12.5)	0	0.02

**Table 2:** Baseline Blood Parameters Between Therapeutic Statin Group vs No Statin Therapy Values are n (%) or mean ± SD. Chi-squared test was used for categorical data, and unpaired Student's t test was used for continuous data between group 1 vs group 2. Values of p < 0.05 were considered statistically significant. HDL, high-density lipoproteins; LDL, low-density lipoproteins; eGFR, estimated glomerular filtration rate.

	Therapeutic Statin (Group 1)	No Statin (Group 2)	P Value
n	56	39	
Total cholesterol, mg/dL	128 ± 24.6	200 ± 41	<0.01
Triglyceride, mg/dL	101 ± 30.5	140 ± 99.2	0.008
HDL cholesterol, mg/dL	43.3 ± 15.1	47.3 ± 16	0.22
LDL cholesterol, mg/dL	68.8 ± 17.3	133 ± 32.9	<0.01
Creatinine, mg/dL	1.15 ± 0.64	0.96 ± 0.57	0.13
eGFR ≤30 mL/kg/min	4 (7.1%)	1 (2.6%)	0.32

Culprit coronary vessel and lesion characteristics

Group 1 patients, compared with group 2, were more likely to have moderate-severe vessel calcification (55.4% vs 10.3% p < 0.01), lesion length greater than 20 mm (51.8% vs 23.1% p = 0.019), stent failure (39.3% vs 7.7%, p < 0.01), and bypass graft failure (23.2% vs 7.7%, p = 0.04). More patients in group 1 compared to group 2 required adjunctive angioplasty techniques and equipment (51.8% vs 7.7% p < 0.01). Results are summarized in Table 3.

**Table 3:** Coronary Lesion and Vessel Characteristics Between Therapeutic Statin Group vs No Statin Therapy at MI Presentation Values are n (%) or mean ± SD. Chi-squared test was used for categorical data, and unpaired Student's t-test was used for continuous data between group 1 vs group 2. Values of p < 0.05 were considered statistically significant. ISR, in-stent restenosis; STEMI, ST-elevation myocardial infarction; NSTEMI, non-ST elevation myocardial infarction; MI, myocardial infarction; CTO, chronic total occlusion; EF, ejection fraction; LAD, left anterior descending; LCx, left circumflex; RCA, right coronary artery; PDA, posterior descending artery; PLB, posterolateral branch; adjunctive angioplasty equipment, defined as using multiple coronary wires, guide extender, thrombectomy, and mechanical circulatory support.

	Therapeutic Statin (Group 1)	No Statin (Group 2)	P Value
n	56	39	
Lesion length >20 mm	29 (51.8)	9 (23.1)	0.019
Vein graft degeneration	13 (23.2)	3 (7.7)	0.04
ISR or stent thrombosis	22 (39.3)	3 (7.7)	< 0.01
Adjunctive angioplasty equipment	29 (51.8)	3 (7.7)	< 0.01
STEMI	9 (16.1)	14 (35.9)	0.02
NSTEMI	47 (83.9)	25 (64.1)	0.02
Moderate-severe calcium deposits	31 (55.4)	4 (10.3)	< 0.01
Excessive proximal vessel tortuosity	1 (1.8)	2 (5.1)	0.35
Vessel angulation >45 degrees	6 (10.7)	2 (5.1)	0.54
Ostial vessel lesion location	3 (5.4)	1 (2.6)	0.50
CTO >3 months	2 (3.6)	0	0.23
EF <40%	20 (35.7)	6 (15.4)	0.02
Single vessel	41 (73.2)	29 (74.4)	0.9
Multivessel	15 (26.8)	10 (25.6)	0.9
Analyzed culprit vessel			
LAD	31 (55.4)	21 (53.8)	0.88
LCx	13 (23.2)	10 (25.6)	0.78
RCA	21 (37.5)	17 (43.6)	0.55
PDA	6 (10.7)	5 (12.8)	0.75
Obtuse marginal	6 (10.7)	5 (12.8)	0.75
Ramus intermedius	0	2 (5.1)	0.08
PLB	4 (7.1)	2 (5.1)	0.69

## Discussion

This retrospective coronary angiographic study of type I MI events demonstrates that, compared to patients not on statin therapy, patients on therapeutic statin therapy (LDL < 100 mg/dL) exhibit culprit lesions that were more often calcified and lengthy, and due to stent failure and bypass graft failure mechanisms. In other words, acute coronary lesions in patients not on statin therapy with elevated LDL (≥100 mg/dL) tend to be more focal, less calcified, and less involved with post-revascularization failure.

The findings in this clinical study echo those of histologic studies evaluating plaques subjected to statin therapy. Statin therapy has been shown to decrease lipid pools, increase the thickness of the fibrous cap, and promote regression of plaque lipid content with increased dense calcium volume [5-8]. This may explain the higher prevalence of calcified lesions in the statin-treated arm (group 1). Furthermore, as statin therapy proves to be effective in managing de novo acute coronary disease, this may have led to a predominance of acute coronary events involving stent or bypass graft failure, as seen in 62.5% of group 1 patients.

This study also highlights several salient features of acute coronary disease in the era of contemporary statin therapy. Firstly, a staggering 62.5% of patients on therapeutic statin therapy (group 1) had acute coronary events from sites of previous revascularization: 39.3% (p < 0.01) from stent failure and 23.2% (p = 0.04) from bypass graft failure. This underscores the limitations of coronary revascularization, including the perpetual risk of stent failure and the limited strategies to reduce vein graft degeneration. Furthermore, it may also be inferred that additional lipid lowering in the statin treatment arm may not necessarily reduce coronary events specific to these target lesion failures, as the mean LDL in the group 1 cohort was already less than 70 mg/dL (68.8 ± 17.3 mg/dL). 

Secondly, these data re-emphasize the importance of optimal medical therapy as part of secondary prevention measures for coronary artery disease. In the group 2 cohort, 33.3% (13 of 39) patients had documented coronary artery disease with a previous history of percutaneous coronary intervention or CABG. All 13 patients were either not compliant or non-tolerant to statin therapy and therefore had untreated dyslipidemia (LDL ≥ 100 mg/dL). Consistent with the over-arching theme, 10 of these 13 non-statins treated patients exhibited an acute coronary lesion due to focal non-calcified lesions which is more consistent with lipid-rich plaque morphology; 1 of the 13 patients had stent thrombosis but may be explained by overall medical non-compliance including antiplatelet agents. As such, with the established role of lipid lowering, the patient should be aggressively counseled about the potential use for PCSK9 inhibitors, especially if demonstrated to be non-tolerant to statin therapy [9].

Lastly, not only are the angiographic features of the acute lesion in group 1 (statin-controlled patients) more advanced compared with group 2, but there was also a signal for heightened procedural complexity, as implicated by the use of adjunctive angioplasty equipment (defined as using multiple coronary wires, guide extender, thrombectomy, and mechanical circulatory support). In this cohort, high calcium burden, longer lesion lengths, friable vein graft degenerations, stent failure, excessive angulation, and tortuosity contribute greatest to vessel complexity. However, one must not misinterpret this as a signal for statins being harmful. Rather, it sheds light into the current-day real-world coronary angioplasty experience, as the phenotype of acute coronary events evolves with lipid lowering. This casts implications to the training and expectations of interventional cardiologists in managing acute lesions that often present with American Heart Association/American College of Cardiology (AHA/ACC) type B or C lesion characteristics.

A limitation to this report stems primarily from being a retrospective study and conducted using patients treated at a single medical center, thereby limiting the generalizability of the findings. Nevertheless, this allowed for consistent data collection and clinical management. Second, there was no routine use of intravascular ultrasound or optical coherence tomography. In particular, stent failure mechanism and aspects of neoatherosclerosis were therefore not able to be determined and details on coronary calcium cannot be specified outside of conventional angiography. Intracoronary imaging may further confirm previous findings of decreased lipid-rich plaque in coronary lesions subjected to statin therapy. Third, the definition of “therapeutic” statin therapy (group 1) and “elevated LDL not on therapy” (group 2) was set at <100 mg/dL and ≥100 mg/dL, respectively, due to relatively small cohort size. In contrast, the 2018 AHA/ACC guidelines recommend a target LDL < 70 mg/dL for patients with clinical atherosclerotic cardiovascular disease (ASCVD) and for individuals with elevated ASCVD risk [10]. Despite this difference in definition, the observation remains that the presentation of acute coronary plaque has shifted with hyperlipidemic therapy with statins.

## Conclusions

In this retrospective study of Type I MI, we demonstrate a significant difference in culprit lesion characteristics in patients on therapeutic statin therapy, compared to patients who are not on statin therapy with an elevated LDL. Acute coronary lesions in the therapeutic statin treated arm are more advanced and are associated with adjunctive angioplasty equipment use. Statin therapy has altered the presentation and management of acute plaque pathophysiology.

## References

[1] Virmani R, Burke AP, Farb A, Kolodgie FD (2006). Pathology of the vulnerable plaque. J Am Coll Cardiol.

[2] Nicholls SJ, Tuzcu EM, Sipahi I (2007). Statins, high-density lipoprotein cholesterol, and regression of coronary atherosclerosis. JAMA.

[3] Hong MK, Park DW, Lee CW (2009). Effects of statin treatments on coronary plaques assessed by volumetric virtual histology intravascular ultrasound analysis. JACC Cardiovasc Interv.

[4] Thygesen K, Alpert JS, Jaffe AS (2018). Fourth Universal Definition of Myocardial Infarction (2018). J Am Coll Cardiol.

[5] Zheng G, Li Y, Huang H, Wang J, Hirayama A, Lin J (2015). The effect of statin therapy on coronary plaque composition using virtual histology intravascular ultrasound: a meta-analysis. PLoS One.

[6] Puri R, Libby P, Nissen SE (2014). Long-term effects of maximally intensive statin therapy on changes in coronary atheroma composition: insights from SATURN. Eur Heart J Cardiovasc Imaging.

[7] Nissen SE, Tuzcu EM, Schoenhagen P (2004). Effect of intensive compared with moderate lipid-lowering therapy on progression of coronary atherosclerosis: a randomized controlled trial. JAMA.

[8] Lee SE, Chang HJ, Sung JM (2018). Effects of statins on coronary atherosclerotic plaques: the PARADIGM study. JACC Cardiovasc Imaging.

[9] Preiss D, Tobert JA, Hovingh GK, Reith C (2020). Lipid-modifying agents, from statins to PCSK9 inhibitors: JACC focus seminar. J Am Coll Cardiol.

[10] Grundy SM, Stone NJ, Bailey AL (2019). 2018 AHA/ACC/AACVPR/AAPA/ABC/ACPM/ADA/AGS/APhA/ASPC/NLA/PCNA guideline on the management of blood cholesterol: a report of the American College of Cardiology/American Heart Association Task Force on Clinical Practice Guidelines. Circulation.

